# Stable Nitrogen Isotope Analysis of Amino Acids by Orbitrap Mass Spectrometry: Application for Extraterrestrial Samples

**DOI:** 10.1002/rcm.10127

**Published:** 2025-08-26

**Authors:** O. M. McIntosh, A. A. Baczynski, M. Matney, H. L. McLain, K. K. Farnsworth, J. P. Dworkin, D. P. Glavin, J. E. Elsila, H. Xie, K. H. Freeman

**Affiliations:** ^1^ Department of Geosciences Pennsylvania State University University Park Pennsylvania USA; ^2^ Solar System Exploration Division NASA Goddard Space Flight Center Greenbelt Maryland USA; ^3^ Catholic University of America Washington District of Columbia USA; ^4^ Center for Research and Exploration in Space Science and Technology, NASA/GSFC Greenbelt Maryland USA; ^5^ Center for Space Sciences and Technology University of Maryland, Baltimore County Baltimore Maryland USA; ^6^ International Center for Isotope Effects Research Nanjing University Nanjing Jiangsu China

**Keywords:** amino acids, carbonaceous chondrites, GC‐Orbitrap‐IRMS, Murchison meteorite, nitrogen stable isotopes

## Abstract

**Background:**

Obtaining isotopic data on soluble organic compounds, such as amino acids, in extraterrestrial samples is crucial for understanding their origins, prebiotic chemistry, and potential contamination. Conventional GC‐IRMS requires grams of material to measure isotopic compositions, limiting the analysis of low‐concentration organics in meteorites and other astromaterials. We present an Orbitrap‐based method optimized for nitrogen isotopic analysis of amino acids.

**Results:**

This method determines δ^15^N values for picomole quantities (< 150 pmol) with 3‰–8‰ precision and accuracy within 2‰ compared with elemental analysis. Our approach was validated using amino acid enantiomer standards and a CM2 Murchison meteorite sample. The Murchison results demonstrate that comparable precision can be achieved on analytes extracted from a total sample size representing less than 7% of the mass previously required for CSIA analysis of the same meteorite.

**Significance:**

These results highlight the potential of Orbitrap mass spectrometry for δ^15^N measurements with less material and lower analyte concentrations. This technique improves our ability to trace the origins and synthetic pathways of amino acids, providing valuable insights into prebiotic chemistry and possible abiotic mechanisms for organic compound formation in primitive solar system materials. Nitrogen isotopes serve as a powerful tool for distinguishing biological from non‐biological sources, aiding in the identification of contamination in meteoritic samples and improving the reliability of analyses involving rare extraterrestrial materials.

## Summary

- GC‐Orbitrap‐IRMS enables accurate nitrogen isotope analysis of amino acids at the picomole level.
- This analytical method achieves picomole sensitivity with a precision of 3‰–8‰ and an accuracy within 2‰.
- GC‐Orbitrap‐IRMS has significant applications for analyzing low mass, low concentration, extraterrestrial samples.
- Nitrogen isotopes can help better distinguish biological from non‐biological sources, aiding in contamination detection.

## 1 Introduction

A significant portion of prebiotic organic material on the early Earth may have been introduced by exogenous sources such as carbonaceous asteroids. Indeed, meteorites contain diverse prebiotic organic compounds, including a variety of proteinogenic and non‐terrestrial amino acids [1], with amino acids serving as the building blocks of proteins and playing essential roles in numerous metabolic processes on Earth. Their functional diversity and ubiquity in biological systems and extraterrestrial materials make them especially attractive candidates to elucidate the origin of life.

Additionally, many amino acids possess an asymmetric center, enabling them to exist as either left‐handed (levorotatory [l]) or right‐handed (dextrorotatory [d]) enantiomers. It is widely believed that the abiotic synthesis of amino acids on early Earth produced racemic mixtures, with l‐ and D‐enantiomers in equal proportions [2, 3]. Biological systems require the almost exclusive selection of either L‐ or D‐enantiomers, yet whether homochirality was a prerequisite for the origin of life or a consequence of it remains an open question. Some amino acids have been found with left‐handed enantiomeric excesses [4, 5, 6], suggesting that meteorites could have contributed to the origin of protein homochirality in life on Earth. However, recent analyses of an aggregate sample returned from asteroid Bennu, considered more pristine than meteoritic material, have found that all non‐protein amino acids detected were racemic within error [7]. These results are particularly important in the quest to understand the origins of homochirality in life on Earth and particularly highlight the challenge to constrain possible misinterpretations from contamination.

To understand how amino acids could have played a role in the origins of life, we must understand how they were formed, a challenging task due to the multiple pathways that can lead to the same chemical structure. These diverse synthesis routes depend on different precursors and reaction processes, both of which can cause varied isotope signatures and thus encode a compound's synthetic history in its isotopic structure [8]. Therefore, analyses of amino acid isotopic composition can provide insights with respect to their origin(s) and mechanisms of formation, shedding light on the prebiotic chemistry that may have been involved in their synthesis, as well as a deeper understanding of the origin of chiral molecules.

The development of gas chromatography/combustion/isotope ratio mass spectrometry (GC‐C‐IRMS) permits such measurements to be made at micromole to nanomole levels for individual amino acids. A few studies [4, 9, 10] were able to obtain carbon, nitrogen, and deuterium isotopic values for several amino acids in different meteorites. However, in most cases, measurements could not be made due to insufficient abundances or chromatographic interferences.

To resolve these issues, emerging methods, such as high‐resolution mass spectrometry (HRMS), show great promise for multi‐element analysis and stable isotope information. Orbitrap‐based Fourier transform mass spectrometers coupled with gas chromatography (GC) or liquid chromatography (LC) have been employed to perform isotopic measurements on various organic compounds, including amino acids and polycyclic aromatic hydrocarbons (PAHs) [11, 12, 13, 14, 15]; but so far, they mainly focus on position‐specific and clumped isotopic analyses of carbon atoms.

Here, we show that another compelling application of Orbitrap‐based measurements lies in its potential to elucidate the origins of amino acids through nitrogen isotopic analysis. Nitrogen isotopes, specifically the relative abundances of ^15^N to ^14^N (expressed as δ^15^N_Air_ values), vary considerably across the solar system. While Earth's atmospheric nitrogen isotopic composition exhibits little variation, typically no more than 2%, extraterrestrial sources display much greater variability, with differences reaching up to 500% [16]. The solar system contains at least three distinct isotopic reservoirs: the protosolar nebula (PSN), highly depleted in ^15^N (δ^15^N_Air_ = −387 ± 8‰), as well as the inner solar system and cometary ices, both enriched in ^15^N relative to the PSN (δ^15^N_Air_ = 0 ± 50‰; δ^15^N_Air_ = 850 ± 150‰, respectively) [16]. This stark contrast makes nitrogen isotopes a powerful tracer for investigating the origins and synthetic pathways of amino acids. Nonetheless, such interpretations require caution, as isotopic mixtures and overlapping sources can complicate the resolution of distinct end‐member signatures.

In primitive meteorites, nitrogen is primarily hosted in organic compounds, and measurements have consistently shown a higher ^15^N enrichment in these materials compared with terrestrial nitrogen [4, 9, 10]. For instance, Engel and Macko [4] observed that the δ^15^N value of a bulk amino‐acid extract from the Murchison meteorite was substantially ^15^N‐enriched (+90‰) compared with biological material on Earth (−20‰ to +30‰) [17]. Moreover, when considering the potential formation pathways of meteoritic amino acids, including Strecker‐cyanohydrin synthesis, Michael addition, CO_2_ addition, and reductive amination of keto acids, isotopic predictions indicate that δ^15^N values would always be higher than those of terrestrial amino acids [9]. This is particularly significant because terrestrial amino acids typically exhibit δ^15^N_Air_ values ranging from −20‰ to +30‰, whereas those measured in meteorites such as Murchison span from +37‰ to +184‰, providing a clear distinction [18]. In contrast, δ^13^C_VPDB_ values for terrestrial amino acids range from −70‰ to +11‰ and overlap with those reported for amino acids in carbonaceous chondrites (−18‰ to +52‰) [18]. As a result, δ^15^N values could be much more sensitive than δ^13^C values, due to the wider range of isotopic ratios and superior discriminatory power, for identifying indigenous extraterrestrial amino acids and assessing potential terrestrial contamination.

In this study, we demonstrate that the Orbitrap‐IRMS can be used to accurately measure the nitrogen isotopic signature of amino acids at very low abundances, ranging from tens to hundreds of picomoles per injection, uniquely allowing ^15^N/^14^N isotope measurements at concentrations currently unmatched by any other instrument. The ability to perform high accuracy (within 2‰ in this study), high precision isotopic measurements on limited quantities of material is crucial for analyzing rare and precious samples, such as extraterrestrial material, where both the total sample mass and the abundance of target organic molecules are restricted. Moreover, while this procedure has valuable applications for investigating kinetic isotope effects, racemization processes, and synthetic pathways of amino acids, its ability to perform high precision nitrogen isotope measurements also provides a means to determine whether amino acids are indigenous to the sample or terrestrial contaminants.

## 2 Materials and Methods

### 2.1 Amino Acid Standards and Extraction of the Murchison Meteorite Sample

L‐valine (USGS73), glycine (USGS64), β‐alanine (99%, Sigma‐Aldrich) and L‐alanine (Arndt Schimmelmann, Indiana University), compounds with known ^15^N/^14^N isotope abundances, were used as standards. L‐valine (USGS75), D‐valine (≥ 98%, Sigma‐Aldrich), glycine (≥ 99%, Sigma‐Aldrich), β‐alanine (99%, Thermo Scientific), L‐alanine (Arndt Schimmelmann, Indiana University), and D‐alanine (≥ 98%, Merck) were treated as unknown samples. Individual stock solutions of each amino acid were prepared by dissolving 0.5 mg of the standard in a solution of water (3 mL, UHPLC grade, Sigma‐Aldrich) and methanol (1 mL, LC–MS grade, Supelco).

To validate the method for the analysis of extraterrestrial materials, amino acids extracted from the CM2 Murchison meteorite, provided originally by the Chicago Field Museum, were also analyzed. The meteorite was prepared at NASA Goddard Space Flight Center (GSFC) following the protocol in Elsila et al. [9]. In summary, the meteorite was prepared in a clean room where it was homogenized into a powder (0.4135 g) prior to hot water extraction at 100°C for 24 h, vapor acid hydrolyzed at 150°C for 3 h, desalted using BIORAD AG50W‐X8 cation exchange columns, and the NH_4_OH eluent collected and dried under vacuum. Two percent of the extracted Murchison sample was kept for amino acid quantification at GSFC using LC mass spectrometry (details in next section), while 98% of the Murchison extract was delivered to Penn State University for isotopic analysis. Two blanks, a procedural solvent blank and a matrix blank (powdered SiO_2_ sample that had previously been combusted in air at 500°C overnight prior to crushing) were prepared in parallel to account for possible contaminants during the crushing and extraction procedures at GSFC.

An in‐house solution of 18 amino acids commonly found in meteorites was prepared at Penn State University to identify the compounds present in the Murchison sample and served as a standard for isotopic measurements. The in‐house amino acid solution contained α‐aminoisobutyric acid (98%, Sigma‐Aldrich), L‐isovaline (95%, AstaTech), D‐isovaline (97%, AmBeed), D‐alanine (≥ 98%, Merck), L‐alanine (Arndt Schimmelmann, Indiana University), D‐valine (≥ 98%, Sigma‐Aldrich), L‐valine (USGS75), DL‐α‐aminobutyric acid (99%, Sigma‐Aldrich), glycine (≥ 99%, Sigma‐Aldrich), β‐alanine (99%, Sigma‐Aldrich), D‐aspartic acid (99%, Sigma‐Aldrich), L‐aspartic acid (≥ 98%, Sigma‐Aldrich), γ‐aminobutyric acid (≥ 99%, Sigma‐Aldrich), D‐glutamic acid (≥ 99%, Sigma‐Aldrich), L‐glutamic acid (USGS40), δ‐amino‐n‐valeric acid (≥ 98%, TCI), and ε‐amino‐n‐caproic acid (≥ 99%, Sigma‐Aldrich). A total of 0.5 mg of each amino acid was diluted in a 4 mL 3:1 H_2_O: methanol stock solution.

### 2.2 LC‐FD/Q‐ToF‐MS Analysis for Amino Acid Quantification in the Murchison Meteorite Sample

Amino acid abundances and distributions were analyzed by LC with fluorescence detection coupled to time‐of‐flight mass spectrometry (LC‐FD/ToF‐MS). The amino acids in the NH_4_OH eluates were derivatized with *o*‐phthaldialdehyde/N‐acetyl‐l‐cysteine (OPA/NAC) followed by their separation and analysis using a Waters ACQUITY Ultra‐Performance Liquid Chromatography system and Waters Xevo G2‐XS QToF‐MS operating in positive ion mode. The extracted, hydrolyzed, desalted samples were dried down with 20 μL of 0.1 M sodium borate before being re‐dissolved in 20 μL of milli‐Q water (< 3 ppt TOC); 5 μL of OPA/NAC derivatization agent was then added and allowed to react for 15 min before being quenched with 75 μL of 0.1 M hydrazine hydrate. After derivatization, 10 μL of the sample was injected, and the C2–C6 amino acids were chromatographically resolved using a Waters BEH C18 column (2.1 × 50 mm, 1.7 μm bead) and a Waters BEH phenyl column (2.1 × 150 mm, 1.7 μm bead) in series. Both columns were maintained at 30°C. The mobile phase conditions for amino acid separations were as follows: flow rate, 150 μL/min; gradient, time in minutes (%B): 0 (0), 35 (55), 45 (100). During the Xevo G2‐XS analysis, a dual electrospray ionization (ESI) system was used for the purpose of implementing lock mass corrections. The primary ESI source was operated using the following parameters: capillary voltage, 3.0 kV; sampling cone voltage, 40 V; cone gas (N_2_) flow, 50 Lhr^−1^, source temperature, 120°C; desolvation gas (N_2_) temperature, 350°C; desolvation gas flow rate, 750 Lhr^−1^.

Mass accuracy was maintained by continuously calibrating via a reference ESI source to supply an independent leucine enkephalin standard signal. The reference ESI source was operated using identical parameters to those used for the primary ESI source, except the reference ESI source used a capillary voltage of 3.0 kV and a reference cone voltage of 30 V. The ToF analyzer was operated in sensitivity mode, which trades off some resolution for a lower limit of detection and a lower limit of quantitation. Even with this tradeoff, the resolution of the lock spray analyte used for this analysis has a resolution of between 20 000 and 28 000 depending on the flow rate used. While the *m/z* of 556.277 for leucine enkephalin is higher than most of the *m/z* used in this analysis, its stability and proximity to the OPA/NAC derivatized analytes allow for a lock mass‐corrected mass accuracy of ±2.5 ppm for most OPA/NAC derivatized analytes, as demonstrated in Glavin and Dworkin et al. [7].

The amino acid abundances and their enantiomeric ratios in the meteorite extracts and controls were determined by comparison of the peak areas generated from the selected ion chromatograms and the UV fluorescence chromatograms (LC‐FD, λ_ex_ = 340 nm, λ_em_ = 450 nm) of their OPA/NAC derivatives to the corresponding peak areas of amino acid standards run under the same chromatographic conditions and included peak identification confirmation by accurate mass using a match tolerance of 10 ppm (ToF‐MS). The reported amino acid concentrations (nmol/g) and D/l ratios (Table 1) are the average values of between three and six separate LC‐FD/ToF‐MS measurements.

**TABLE 1:** Summary of the D/l ratios and average abundances (nmol/g) of the two‐ to six‐carbon amino acids in the 6 M HCl‐hydrolyzed (total) water extracts of the CM2 Murchison meteorite measured by LC‐FD/Q‐ToF‐MS^a^.

Carbon #	Amino acid	D/L ratios	Abundances (nmol/g)
2	Glycine	n/a	52.0 ± 0.4
3	D‐alanine	0.78 ± 0.02	3.6 ± 0.1
	L‐alanine		4.4 ± 0.1
	β‐alanine	n/a	17.2 ± 0.3
	D‐serine	0.30 ± 0.01	0.17 ± 0.002
	L‐serine		0.28 ± 0.01
4	D‐aspartic acid	0.59 ± 0.02	0.59 ± 0.01
	L‐aspartic acid		0.94 ± 0.02
	D,L‐α‐amino‐*n*‐butyric acid	n/a	3.1 ± 0.04
	D‐β‐amino‐*n*‐butyric acid	1.27 ± 0.03^c^	4.6 ± 0.04
	L‐β‐amino‐*n*‐butyric acid		3.7 ± 0.1
	γ‐amino‐*n*‐butyric acid^b^	n/a	6.5 ± 0.1
	α‐amino isobutyric acid	n/a	15.8 ± 0.2
5	D‐glutamic acid	0.39 ± 0.01	2.2 ± 0.1
	L‐glutamic acid		5.5 ± 0.1
	D‐valine	0.43 ± 0.01	0.57 ± 0.01^c^
	L‐valine		1.3 ± 0.01^c^
	D‐isovaline	0.82 ± 0.01	8.0 ± 0.055
	L‐isovaline		9.7 ± 0.055
	D‐norvaline	n/a	< 0.1
	L‐norvaline	n/a	< 0.1
6	ε‐amino‐*n‐*caproic acid	n/a	1.4 ± 0.02

^a^ Extracts were analyzed by OPA/NAC derivatization and LC‐FD/Q‐ToF‐MS. For the LC‐FD/Q‐ToF‐MS data, the mono‐isotopic masses of each protonated OPA/NAC amino acid derivative (M^+^ H^+^) were used for quantification, and final peak integrations included background level correction using a procedural blank and a comparison of the peak areas with those of an amino acid standard run on the same day. The uncertainties (Δx) are based on the standard deviation of the average value of 3–6 separate measurements (n) with a standard error, Δx = σx · (n)^−1/2^.

^b^ The amino acids γ‐aminobutyric acid and β‐amino‐*n*‐isobutyric acid coelute with the conditions used for this analysis.

^c^ Abundance values for these analytes have a greater uncertainty due to possible coelutions using the LCMS gradient optimized for most C2–C6 amino acids, opposed to that specifically designed for the C5 amino acids.

Abbreviation: n/a: not applicable because the amino acid is non‐chiral or present at too low an abundance.

### 2.3 Derivatization for GC‐Orbitrap‐IRMS Measurements

The amino acids were derivatized by methylation of the carboxyl group and trifluoroacetylation of the amine group, producing N,O‐bis(trifluoroacetyl) methyl esters (protocol adapted from Corr et al. [19]). Derivatization of the amino acids is needed to increase their volatility for GC separation. For the amino acid standards, 200 μL of the stock solution was transferred into 2 mL GC vials and dried down under a high purity (> 99.99%) N_2_ flow. The Murchison extract, procedural blank, and powdered SiO_2_ samples were already dried at GSFC when received.

For the first step of the derivatization, 100 μL of methanol was added to each vial and placed on ice; acetyl chloride (25 μL; ≥ 99.0%, Sigma‐Aldrich) was added dropwise. Vials were capped and heated at 80°C for 1 h. The resulting methyl esters were dried under N_2_ at room temperature. Hexane (120 μL, 99%, Supelco) and trifluoroacetic anhydride (TFAA) (60 μL; ≥ 99.0%, Supelco) were added to each vial and heated at 70°C for 30 min. Excess reagent was removed under N_2_ at room temperature. The derivatized standards were suspended in 1 mL of hexane for GC analyses, while the Murchison extract, powdered silica sample, and procedural blank were suspended in 100 μL of hexane.

To minimize contamination, all glassware and tools were either baked in an oven at 460°C for 8 h or solvent‐rinsed using a sequential cleaning procedure of methanol, dichloromethane (≥ 99.8%, Supelco), and hexane. A derivatization blank was prepared in parallel to account for potential contamination and derivatization byproducts.

### 2.4 GC‐Orbitrap‐IRMS Analysis and Peak Trapping System for Isotopic Measurements

Isotope analyses of amino acid mass spectral fragments were performed on a Q‐Exactive Orbitrap mass spectrometer (Thermo Scientific) with samples introduced via a TRACE 1310 GC (Thermo Scientific) equipped with a split/splitless injector. Direct measurements of analytes eluting from the GC column allow for the optimization of chromatography and identification of peak retention time. However, this approach is limited by the number of ions observed per scan in the Orbitrap, the duration of chromatographic peak elution from the GC [20], the rarity of ^15^N and chromatographic fractionation. To alleviate these issues, our instrumental setup was modified to allow high‐precision isotope analysis, employing a peak trapping technique to enhance analytical accuracy and precision.

**FIGURE 1:**
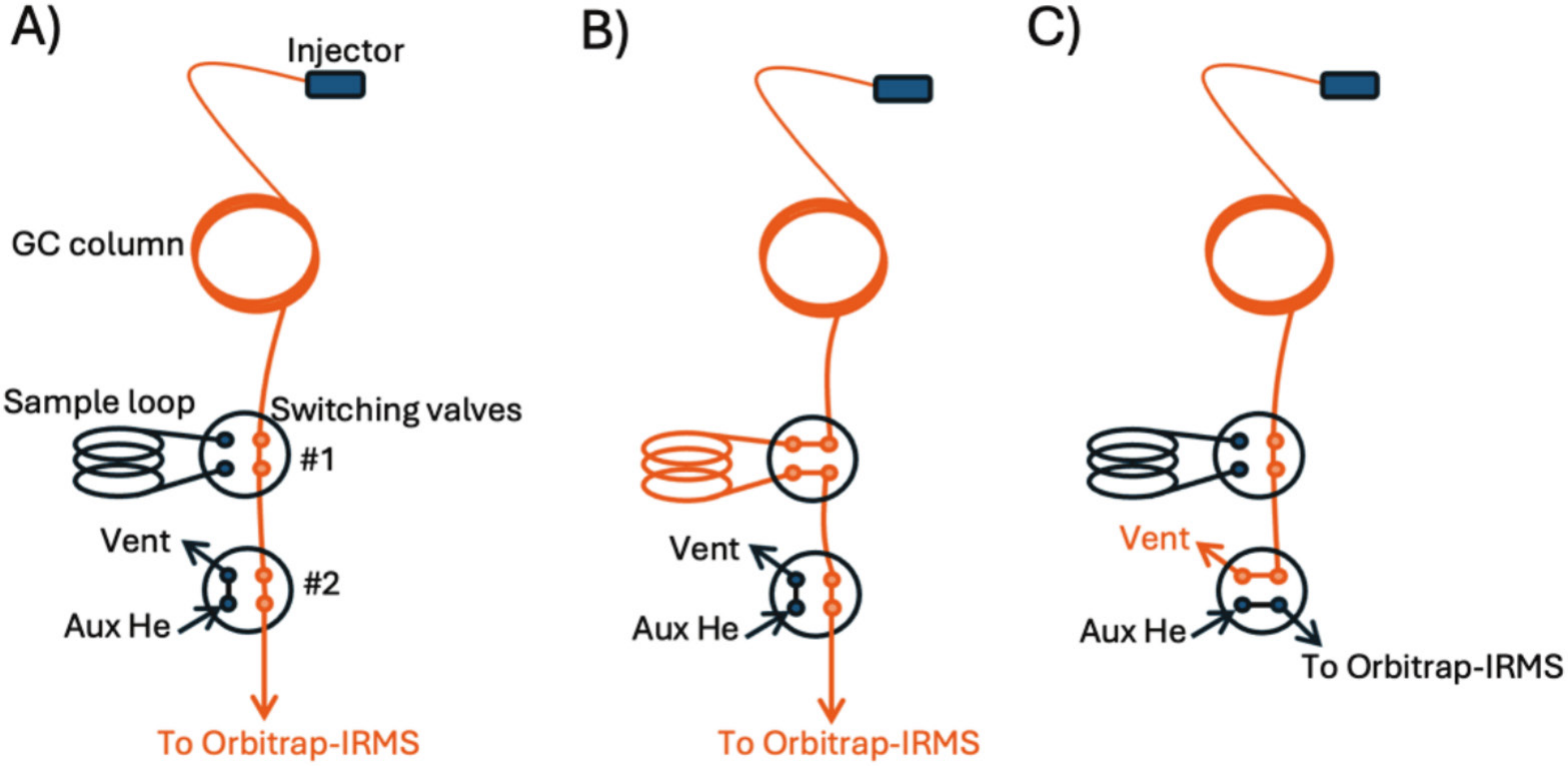
Schematic of the GC‐Orbitrap‐IRMS instrumental setup: (A) GC–Orbitrap‐IRMS mode, (B) the GC–SL–Orbitrap‐IRMS mode, and (C) He–MS mode.

The system includes a customized sample‐trapping setup located within the GC oven, between the GC column and the MS transfer line. This setup incorporates a 10 mL stainless steel sample loop (SL) (VICI; SL10KCSTP) deactivated with SilcoNert 2000 by SilcoTek to prevent chemisorption or degradation of samples and two 4‐port valves (VICI‐Valco Instruments Co. Inc.; A6N4WT) actuated by pneumatic solenoids (Humphrey 310‐24‐VDC). These solenoids are controlled by the “external events” function of the Aux Temperature/Cryo Module of the Trace 1310 GC whose operation is incorporated into the method for higher precision and repeatability. By changing the positions of the two switch valves, the system can be configured into three modes: GC–Orbitrap‐IRMS, GC–SL–Orbitrap‐IRMS, and He–MS (Figure 1). The GC–Orbitrap‐IRMS mode is used for direct elution (Figure 1A), the GC–SL–Orbitrap‐IRMS mode is used for trapping the analyte into the SL for isotopic measurements (Figure 1B), and the He–MS mode (Figure 1C) is used to maintain system integrity, such as vacuum pressure in the Orbitrap‐IRMS, during routine maintenance or column changes.

During analysis, the system first operates as a standard GC–MS (GC–Orbitrap‐IRMS mode; Figure 1A). The SL is isolated from the flow path during this phase. As the target analyte elutes from the GC column, the valve switches to direct the analyte into the SL (GC–SL–Orbitrap‐IRMS mode; Figure 1B). The analyte is then trapped and allowed to diffuse within the loop in its volatile form for 15–20 min or longer depending on the GC run length. This equilibrating period prior to peak release ensures symmetrical, high‐quality peaks and mixing; thus, mitigating issues related to chromatographic fractionation [11]. After trapping the targeted analyte, the system reverts to GC–Orbitrap‐IRMS mode to allow all the other analytes to elute out of the column. Finally, the valve switches back to GC–SL–Orbitrap‐IRMS mode at the end of the run, and the trapped analyte is slowly eluted into the Orbitrap‐IRMS for extended analysis, resulting in a broad peak, maximizing the number of scans, and providing up to 10 min of analysis time (instead of 10–30 s using the direct elution method).

Derivatized amino acid extract was injected in splitless mode at 250°C with a 4 mm ID deactivated glass liner. Chromatographic separation was carried out on a 50 m × 0.25 mm ID capillary column (LIPODEX E; Macherey‐Nagel) with a 1.0 mL/min helium carrier gas flow. The GC oven temperature program was set to start at 60°C and ramp up 2°C/min up to 100°C, followed by a 4°C/min ramp up to 200°C, held for 20 min.

The transfer line and ion source temperatures of the MS were set at 250°C and 280°C, respectively. Ions produced by electron impact (energy of 70 eV) were scanned between mass‐to‐charge ratios (*m/z*) of 30–300 in direct elution mode. To maximize sensitivity for the fragment ions of interest, we employed focused mass window measurements in peak trapping mode, using a 10 Da scan range centered around the mass of the unsubstituted molecular ion of each fragment (Table 2). This approach reduces the number of ions observed per acquisition so that each measurement is dominated by the target ion fragment while minimizing the contribution from background and contaminant ions. A nominal mass resolution (m/Δm) of 60 000 at *m/z* 200 was used across all analyses, and the automatic gain control (AGC) target was set to 200 000 based on the relative abundance of the target fragments.

**TABLE 2:** Structures of the derivatized amino acids with the targeted fragment highlighted in orange. For each amino acid, the scan range and exact masses of the base peak (unsubstituted isotopologue) and the corresponding ^15^N‐ and ^13^C‐substituted ions are listed.

Amino acids	Structure and targeted fragment	Scan range (*m/z*)	Unsubstituted isotopologue (*m/z*)	^15^N‐bearing isotopoloque (*m/z*)	^13^C‐bearing isotopologue (*m/z*)
Glycine	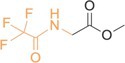	121–131	126.01612	127.01334	127.01943
β‐alanine	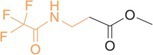	134–144	139.02400	140.02130	140.02744
DL‐alanine	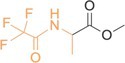	135–145	140.03178	141.02901	141.03513
DL‐valine	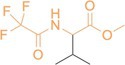	163–173	168.06299	169.06018	169.06630
α‐AIB	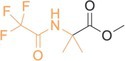	149–159	154.04742	155.04457	155.05069

The scan ranges, resolution, and AGC targets were optimized to ensure that, under the selected experimental conditions, most of the detected ions originated from the compounds of interest [11]. These settings were also chosen to mitigate space‐charge effects, a phenomenon in which excessive ion populations in the C‐trap or Orbitrap interact, disrupting the stable, harmonic orbits essential for accurate Fourier‐transform mass spectrometry [11, 21]. Such effects can be reduced by narrowing the mass scan window or lowering the AGC target to limit the number of extraneous ions that might interfere with the measurement of isotope ratios. Additionally, reducing the resolution shortens the time ions spend in the Orbitrap, decreasing the likelihood of ion‐ion interactions and orbit instability, while generally enhancing sensitivity and reducing the impact of contaminants on isotope ratio accuracy [21].

Before analyses of each amino acid using the peak trapping mode, we performed direct elution measurements of samples and standards to confirm the trapping window based on the retention time of each compound of interest and detect any potential extraneous ions that co‐eluted with analyte fragments within the mass window of interest.

### 2.5 Nitrogen Isotopic Measurements

Candidate fragment ions were identified from the full mass spectrum (*m/z* 30–300) of derivatized amino acids. The molecular structures of candidate fragment ions were elucidated by examining their exact masses in combination with predictions from spectral interpretation software (NIST MS Interpreter). The measured fragment ions have monoisotopic peaks, with an associated ^15^N‐substituted signal, allowing us to measure the isotope ratio of a fragment directly. The ^15^N fragments of the targeted amino acids were baseline resolved from the ^13^C‐bearing isotopologues (Figure S1), ensuring that relative peak intensities remained unaffected. Additionally, the data showed no evidence of coalescence, defined here as a shift in the measured mass difference between two minor species relative to their expected mass difference, caused by space‐charge effects [11] (Table 2).

To determine the nitrogen isotopic ratio of each amino acid, we selected the following fragments (base peak): *m/z* 168 Da for L‐ and D‐valine, *m/z* 126 Da for glycine, *m/z* 140 Da for L‐ and D‐alanine, *m/z* 139 Da for β‐alanine, and *m/z* 154 Da for α‐aminoisobutyric acid (Table 2).

Five replicate analyses were performed for each standard and sample. Standards were analyzed between sample runs under identical analytical conditions to monitor instrument stability and variability. This strategy is consistent with the framework proposed by Eiler et al. [11], whereby instrumental mass fractionation effects are comparable for chemically similar compounds, enabling reliable standardization across measurements.

Additionally, to minimize analytical bias and improve measurement accuracy, standard concentrations were adjusted to closely match the absolute intensity of the corresponding sample in the SL. This approach follows the principle of identical treatment, which conveys that isotope ratio measurements are most accurate when differences between the sample and reference, particularly in injection volume, concentration, and measurement conditions, are minimized. By ensuring that both sample and standard are subjected to as identical conditions as possible, it reduces potential sources of analytical error and improves the reliability of isotope ratio comparisons by controlling for instrumental mass fractionation [11].

The chromatograms obtained from each acquisition were then used to calculate isotope ratios. FT Statistic software (Thermo Fisher) was used to extract the ion intensities, injection time (IT), total ion current (TIC), and other acquisition parameters from the .RAW Orbitrap data files. The data were then processed with a Python script available on the Caltech data repository [22], which converts ion intensities to ion counts using Equation (1) as described in Eiler et al. [11]:

(1) $$N_{\mathrm{io}}=\frac{S}{N}*\frac{C_{N}}{z}*\sqrt{\frac{R_{N}}{R}}*\sqrt{\mu }$$

where N_io_ is the number of observed ions, S is the reported signal intensity for the target molecular ion, N is the noise associated with that signal, both measured at the formal resolution setting R (defined at *m/z* 200). C_N_ is an experimentally determined constant representing the number of charges corresponding to the noise band at a reference resolution R_N_. z is the charge per ion at the mass of interest, and μ is the number of micro‐scans (i.e., sequential ion packet injections analyzed and averaged within a measurement cycle to produce a single scan).

For each acquisition, the data files were reviewed to identify a consistent integration window during which the Orbitrap mass analyzer operated under stable AGC. This window was defined by selecting periods when the ion IT remained below the instrument's maximum IT setting (200 ms) and isotope ratios (^15^N/^14^N) were stable across the acquisition. As an additional check to confirm stable AGC, we verified that the variation in the product of TIC and IT remained within 20% across all scans used for isotope ratio analysis. Acquisitions that were not under these conditions were discarded.

Each acquisition contained thousands of scans reporting signals for the monoisotopic (^14^N) fragment, the ^15^N‐substituted isotopologue, or both. The Python code sums the ion counts for each isotopologue on the entire chromatographic peak and outputs the ^15^N/^14^N isotope ratio for each standard and sample replicate. The relative difference in isotope ratio between sample and standard was then expressed using the conventional delta (δ) notation, reported in parts per thousand (permil; ‰) (Equation 2). For each fragment, the final delta value represents the average of the delta values obtained for each set of replicate measurements (*n* = 5) (Equation 3).

(2) δ15N=Rsample15Rstandard15−1×1000

(3) δNaverage=∑δN15n15

The standard error (SE) for the averaged δ^15^N values was calculated as follows (Equation 4):

(4) $$\mathrm{SE}=\frac{S}{\sqrt{n}}$$

R is the observed isotopic ratio; n is the number of replicates of the measurement, and S is the standard deviation.

### 2.6 Conversion Into International Reference Scale (Air)

To contextualize our results within the already‐established framework of the stable isotope geochemistry of nitrogen, we converted the δ^15^N values measured within our in‐house standard reference frame into the more widely used “primary standard” atmospheric (air) nitrogen isotope scale [23].

The nitrogen isotopic ratios of our in‐house amino acid references were characterized by elemental analysis–isotope ratio mass spectrometry (EA‐IRMS) (see Supporting Information for method), allowing us to anchor the δ^15^N values on the international scale. To perform the conversion from our in‐house standard reference frame to the atmospheric reference frame, we added the measured δ^15^N_Air_ value of nitrogen as measured by EA/IRMS to our measured nitrogen delta value in the house standard reference frame (Equation 5). The last term of the equation is a second‐order correction that accounts for the multiplicative relationship between δ isotopic values referenced to different standards, ensuring accuracy, especially when δ values are large.

(5) $$\begin{array}{c} \delta^{15}N_{\text{sample},\mathrm{Air}}=\delta^{15}N_{\text{sample},\text{standard}}+\delta^{15}N_{\text{standard},\mathrm{Air}}\\ \;+\left(\frac{1}{1000}\right)\;\left(\delta^{15}N_{\text{sample},\text{standard}}\right)\;\left(\delta^{15}N_{\text{standard},\mathrm{Air}}\right) \end{array}$$

## 3 Results

### 3.1 Standards

The accuracy of the isotopic measurements in this study was validated by comparing the δ^15^N_Air_ values of amino acid standards obtained with the GC‐Orbitrap‐IRMS with those from conventional EA/IRMS, using the international isotopic reference scale to enable direct comparison of isotope abundances.

For each measurement, approximately 330, 280, 215, and 310 pmol of nitrogen were injected for glycine, alanine, valine, and β‐alanine standards, respectively. The Orbitrap δ^15^N_Air_ values obtained for all the amino acids tested agree within error with the certified values and those obtained using conventional EA/IRMS (Table 3). The consistency of our values with the independently measured EA‐IRMS δ^15^N values and certified standards also suggests that any instrumental fractionation effects are effectively canceled and validates the absolute reference frame of our study.

**TABLE 3:** δ^15^N_Air_ values and standard error of pure standards measured with the Orbitrap‐IRMS instruments for glycine, D‐ and L‐alanine, D‐ and L‐valine, and β‐alanine compared with δ^15^N_Air_ certified and EA/IRMS values.

	Glycine (USGS64)	L‐alanine	D‐alanine	L‐valine (USGS75)	D‐valine	β‐alanine	α‐AIB
Certified	1.76 ± 0.06	43.25 ± 0.07	n/a	61.53 ± 0.14	n/a	n/a	n/a
Orbitrap‐IRMS	−0.4 ± 3.2	41.9 ± 7.7	−3.4 ± 5.7	60.1 ± 3.5	4.2 ± 2.8	−14.6 ± 4.0	n/a^a^
EA/IRMS	1.32 ± 0.02	43.22 ± 0.02	−1.05 ± 0.03	62.0 ± 0.2	5.88 ± 0.01	−14.1 ± 0.02	8.97 ± 0.04

^a^ Only the EA/IRMS value is reported for alpha‐AIB, which served as the reference standard in the Murchison meteorite measurments (see Section 3.2).

While the δ^15^N range spanned by the certified standards is narrower than that of extraterrestrial amino acids, this range establishes scale accuracy over terrestrial values. Extrapolation beyond this range to the higher δ^15^N values observed in extraterrestrial samples introduces a potential for scale compression, though at enrichments of ~50‰ the effect is expected to be negligible relative to the much larger ranges observed in meteoritic organics. Nonetheless, we acknowledge this as a caveat and note that future work incorporating standards with higher δ^15^N values would better constrain the magnitude of any possible scale non‐linearity at these extreme enrichments.

The shot noise limit is the fundamental statistical fluctuation in signal intensity caused by the random nature of ion detection, playing a crucial role in determining an instrument's precision and defined as follows:

(6) $$\sigma_{\delta }^{2}=2\times 10^{6}{\left(\frac{\sigma_{R}}{R}\right)}^{2}=2\times 10^{6}\frac{{\left(1+R\right)}^{2}}{\mathrm{Em}N_{A}R}=2\times 10^{6}\;\left(\frac{1}{N_{M}}+\frac{1}{N_{m}}\right)$$

where *σ*_*δ*_ is the standard deviation of replicate measurements of *δ*, *R* is the ion current ratio, *EmN*_*A*_ is the number of ions collected, and *N*_*M*_ and *N*_*m*_ are the number of major and minor isotopologue ions collected, respectively. See Merritt and Hayes [24] and Hayes [25] for a complete derivation of shot noise statistics.

When comparing the Orbitrap‐IRMS to the EA/IRMS, a significantly lower amount of analyte was injected (< 500 pmol vs. > 10^7^ pmol), resulting in much lower ion counts (10^6^ vs. 10^12^) (Equation 6) for each amino acid. The lower number of ions collected by the Orbitrap‐IRMS increases its shot noise limit, ultimately reducing its measurement precision.

### 3.2 Murchison Meteorite

**FIGURE 2:**
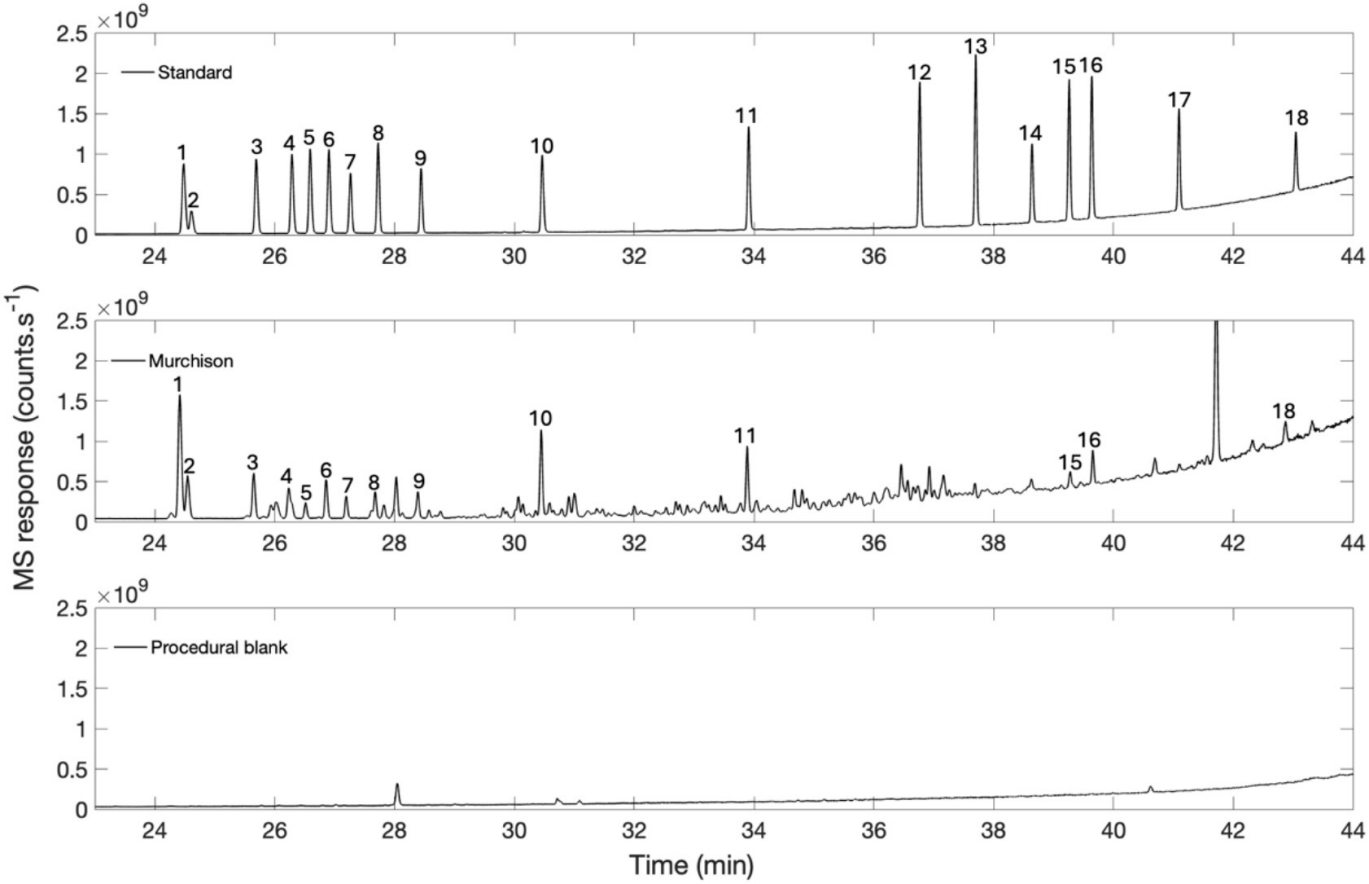
Chromatograms of the derivatized amino acid standards (top), Murchison (middle), and procedural blank (bottom) obtained with the GC‐Orbitrap‐IRMS mode. (1) α‐aminoisobutyric acid, (2) L‐isovaline, (3) D‐isovaline, (4) D‐alanine, (5) D‐valine, (6) L‐alanine, (7) D‐α‐aminobutyric acid, (8) L‐valine, (9) L‐ α‐aminobutyric acid, (10) glycine, (11) β‐alanine, (12) D‐aspartic acid, (13) L‐aspartic acid, (14) γ‐aminobutyric acid, (15) D‐glutamic acid, (16) L‐glutamic acid, (17) δ‐amino‐*n*‐valeric acid, (18) ε‐amino‐*n*‐caproic acid. Unlabeled peaks were unidentified.

The Murchison meteorite extract contained 14 of the 18 amino acids above detection limit present in the prepared standard amino acid mix. D‐aspartic acid and ε‐amino‐*n*‐caproic acid were either not present or were below the detection limit of our instrument (< 10 pmol). The procedural blank (Figure 2, bottom chromatogram) showed no detectable amino acids, indicating the absence of significant contamination imparted by the extraction and derivatization procedures. The three most abundant amino acids in the Murchison extract, α‐aminoisobutyric acid, glycine, and β‐alanine (Figure 2, Peaks 1, 10, and 11, respectively), were selected for nitrogen isotopic measurements. While not tested here due to a limited amount of sample, other peaks could have been analyzed by injecting a larger amount of the sample.

For each GC‐Orbitrap‐IRMS measurement, approximately 430, 142, and 130 pmol of nitrogen were injected for glycine, β‐alanine, and α‐aminoisobutyric acid, respectively, as determined by the quantification of the amino acid by LC‐FD/ToF‐MS (Table 1). The concentration of glycine measured by LC‐FD/ToF‐MS (52.0 nmol/g) is significantly higher than the concentrations measured for β‐alanine and α‐aminoisobutyric acid (17.2 and 15.8 nmol/g, respectively). However, these differences in concentration are not reflected in the chromatogram of the Murchison sample (Figure 2), where glycine appears to be present in approximately the same amount as β‐alanine and α‐aminoisobutyric acid. These discrepancies could be explained by less effective derivatization of certain molecules [26]; in this case, glycine. Consequently, the actual amount of nitrogen injected is likely lower than calculated.

**FIGURE 3:**
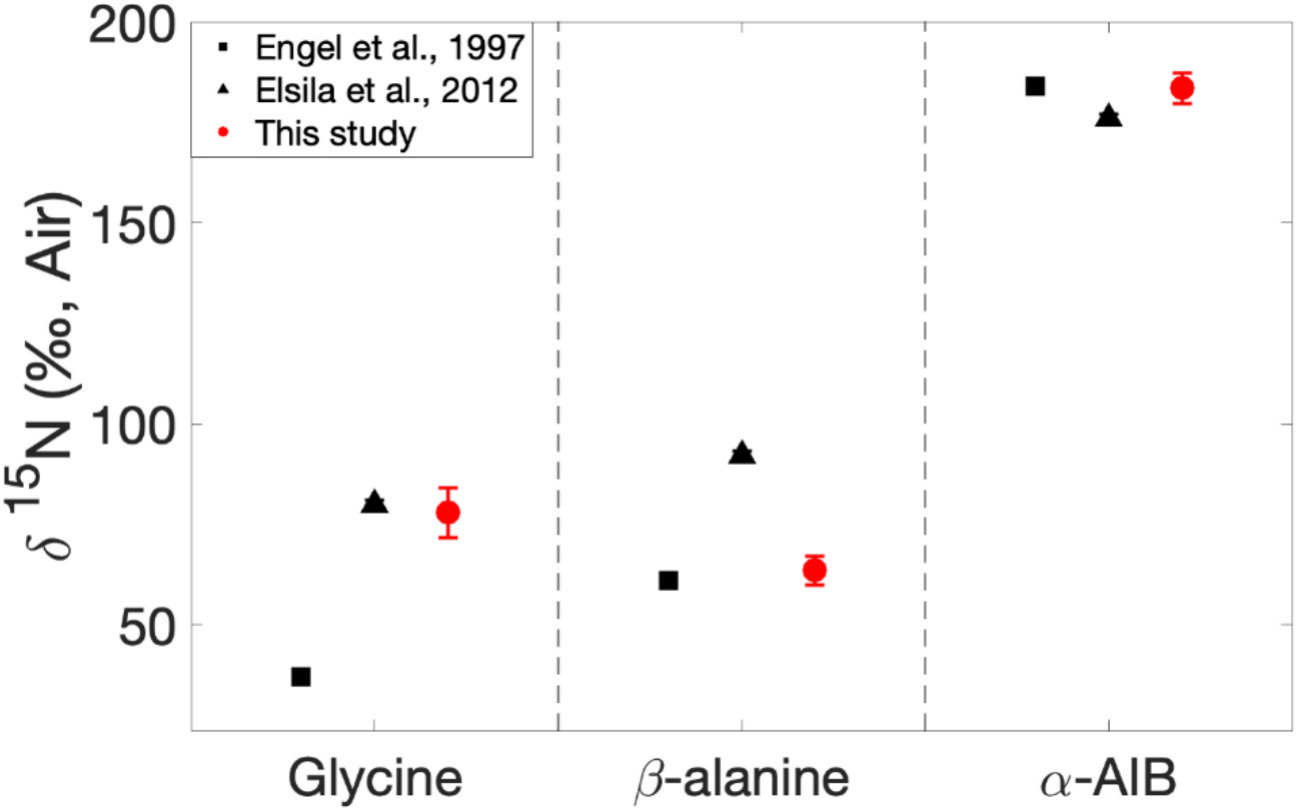
Plot of δ^15^N (‰, Air) values obtained for glycine, β‐alanine, and α‐aminoisobutyric acid (α‐AIB) using the Orbitrap‐IRMS (red dots), compared with values from studies by Engel and Macko [4] (black squares) and Elsila et al. [9] (black triangles) on the Murchison meteorite.

The nitrogen isotopic values obtained for glycine (77.9 ± 6.1‰), β‐alanine (63.5 ± 3.6‰), and α‐aminoisobutyric acid (183.5 ± 3.9‰) extracted from the Murchison meteorite are consistent with an extraterrestrial origin, as they are significantly ^15^N‐enriched compared with terrestrial organic matter, which typically ranges from −20‰ to +30‰ [18]. These isotopic values are in agreement with those reported in previous studies of the Murchison meteorite [4, 9] (Figure 3), with over 15 times less material (7.3 g [4] and 6.3 g [9] vs. 0.4 g in this study). The α‐aminoisobutyric acid δ^15^N_Air_ values match those reported by both Elsila et al. [9] and Engel and Macko [4]. On the other hand, our Orbitrap‐IRMS values for glycine and β‐alanine are consistent with Elsila et al. [9] and Engel and Macko [4], respectively, but not both. This discrepancy might reflect the heterogeneous nature of the Murchison meteorite [27, 28, 29]. Overall, these results further support the accuracy of the isotopic values obtained with the Orbitrap‐IRMS and the robustness of the analytical method presented.

## 4 Discussion and Conclusion

The ability to measure the nitrogen isotopic composition of amino acids at low concentrations using the GC‐Orbitrap‐IRMS instrumental setup presented in this study represents a significant advancement in the field of geochemistry and astrobiology. This study has demonstrated that the Orbitrap‐IRMS trap can uniquely achieve ^15^N/^14^N isotope measurements at concentrations that are currently unmatched by other instruments and for complex extraterrestrial samples such as the Murchison meteorite. This high sensitivity not only allows for the analysis of amino acids in much lower abundances but also expands the range of amino acid targets, particularly enantiomers, and samples that can now be measured. This is a crucial advancement, given that amino acids are difficult or impossible to detect with conventional methods due to their trace amounts. The high resolution of the Orbitrap‐IRMS offers additional benefits, particularly its potential to resolve coeluting peaks [21]. This capability, though not demonstrated in the current study, is significant as it could enable precise isotopic measurements of nitrogen even in complex mixtures if the ion fragments within the coeluting peak have different mass to charge ratios.

Moreover, we argue that nitrogen isotopes could serve as a more effective indicator for detecting potential terrestrial contamination than the carbon isotopes typically used in meteoritic sample analysis. Indeed, δ^15^N values for extraterrestrial amino acids are usually 50‰–200‰ higher than those of terrestrial amino acids, whereas δ^13^C values are rarely more than 50‰ [4, 9]. These stark isotopic differences could better help with identifying potential contaminants in a sample using a mass balance calculation.

In conclusion, the application of Orbitrap‐IRMS to measure nitrogen isotopes in amino acid enantiomers holds great promise for advancing our understanding of the origins and synthetic pathways of amino acids, both terrestrial and extraterrestrial. The ability to accurately measure δ^15^N values at the picomole level opens new avenues for exploring the complex history of amino acids and offers a powerful tool for distinguishing between biological and non‐biological sources and identifying potential contaminations in meteoritic samples.

## Author Contributions

**O.M. McIntosh:** investigation, writing – original draft, methodology, validation, visualization, writing – review and editing, conceptualization, formal analysis. **A.A. Baczynski:** investigation. **M. Matney:** investigation. **H.L. McLain:** investigation, writing – original draft, methodology. **K.K. Farnsworth:** investigation, writing – original draft, methodology. **J.P. Dworkin:** project administration, supervision. **D.P. Glavin:** project administration, supervision. **J.E. Elsila:** project administration, supervision. **H. Xie:** writing – review and editing. **K.H. Freeman:** conceptualization, funding acquisition, methodology, project administration, supervision, resources.

## Peer Review

The peer review history for this article is available at https://www.webofscience.com/api/gateway/wos/peer‐review/10.1002/rcm.10127.

## Supporting information


**Figure S1:** Mass windows of measured mass spectral fragments.

## Data Availability

The instrument data supporting the experimental results in this study will be available at https://doi.org/10.26208/KZB5‐CV48.
