# Supplementary material for: Stable Nitrogen Isotope Analysis of Amino Acids by Orbitrap Mass Spectrometry: Application for Extraterrestrial Samples
Source: Rapid Commun Mass Spectrom. 2025 Aug 26;39(23):e10127. doi: 10.1002/rcm.10127 (PMC12379112; doi:10.1002/rcm.10127)
Supplement: Supplementary file 1 — Figure S1: Mass windows of measured mass spectral fragments. [file RCM-39-e10127-s001.docx]

**Supplementary information:**

**Stable nitrogen isotope analysis of amino acid by Orbitrap mass spectrometry: application for extraterrestrial samples**

O.M. McIntosh^1*^, A.A. Baczynski^1^, M. Matney^1^, H.L. McLain^2,3,4^, K.K. Farnsworth^2,3,5^, J.P. Dworkin^2^, D.P. Glavin^2^, J.E. Elsila^2^, H. Xie^1,6^, K.H. Freeman^1^

^1^Department of Geosciences, Pennsylvania State University, University Park, PA, USA. *Email corresponding author: [omm5285@psu.edu](mailto:omm5285@psu.edu).  ^2^ Solar System Exploration Division, NASA Goddard Space Flight Center, Greenbelt, MD, USA.^3^ Center for Research and Exploration in Space Science and Technology, NASA/GSFC, Greenbelt, MD, USA. ^4^The Catholic University of America, Washington DC, USA. ^5^Center for Space Sciences and Technology, University of Maryland Baltimore County, Baltimore, MD, USA.^6^ International Center for Isotope Effects Research, Nanjing University, Nanjing, Jiangsu Province, China.

**
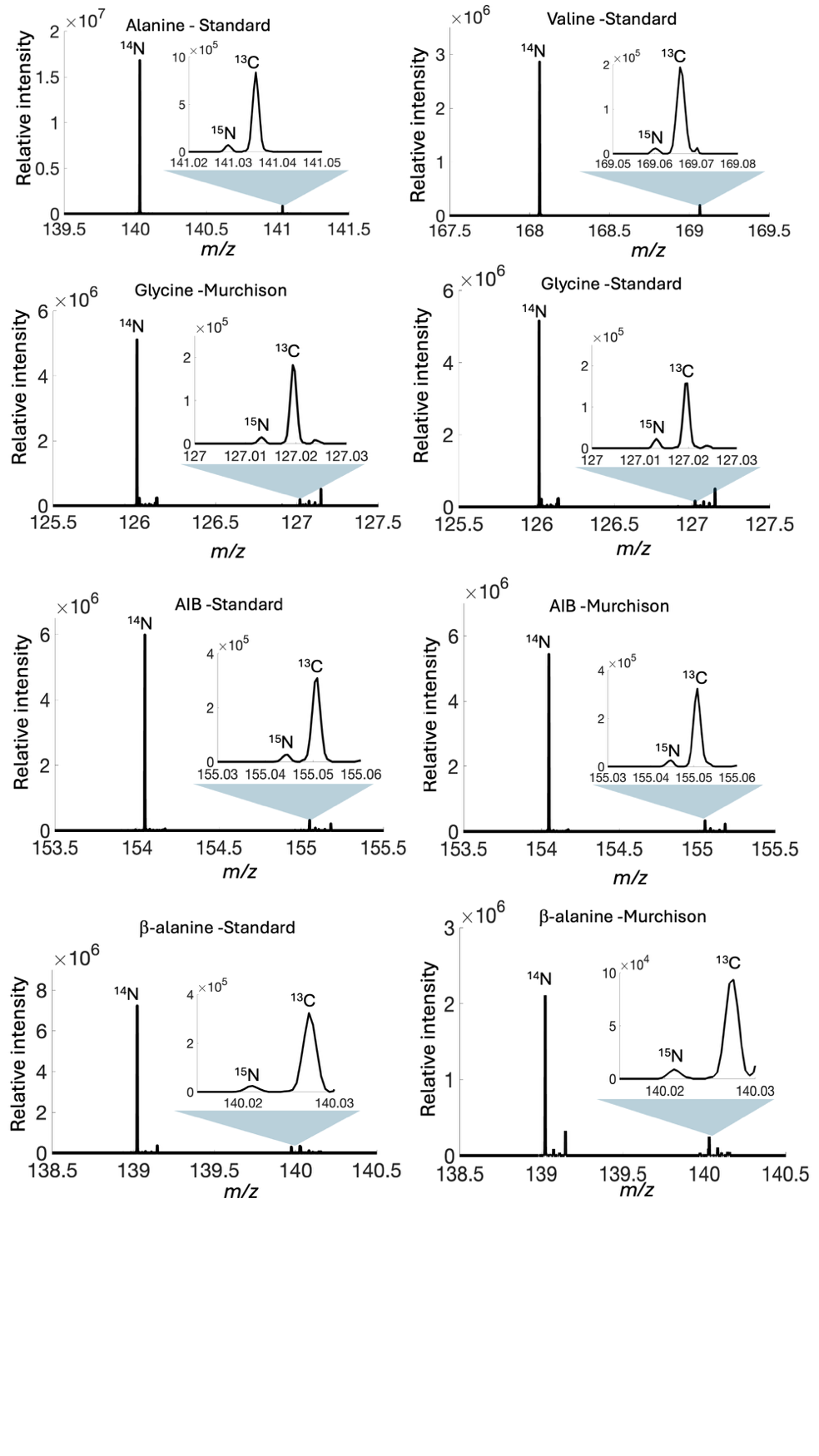
Figure S1:** Mass windows of measured mass spectral fragments.

**EA/IRMS method**

Nitrogen isotope ratios were measured in triplicate using a Costech ECS 4010 combustion elemental analyzer coupled to a Thermo Delta V Plus isotope ratio mass spectrometer. Nitrogen isotope values are reported in delta notation normalized to the international standard atmospheric air (AIR) using the International Atomic Energy Agency (IAEA, United Nations, Vienna, Austria) reference standard N_2_ (ammonium sulfate) and US Geological Survey (USGS, Reston, VA, USA) reference standard 25 (ammonium sulfate). Replicate measurements of the two calibration standards and an in-house caffeine standard that was evaluated as an unknown indicate a measurement precision of < ±0.1‰ (n = 21; 1σ).

**Isotope linearity**

For precise and accurate isotope ratio measurements using conventional IRMS, sample isotopic compositions are determined relative to a known standard. This same approach is employed in Orbitrap-based isotope ratio mass spectrometry (IRMS) to improve measurement accuracy and precision. Since analyte concentrations in unknown samples can vary, achieving perfectly matched selected ion current (SIC) intensities isn’t always possible. In isotope ratio measurements by mass spectrometry, differences in ion current intensity can introduce errors in the measured isotope ratios if not properly corrected. To optimize the reliability of these comparisons, the principle of *identical treatment* is followed, ensuring that both the sample and the standard are analyzed under as similar conditions as possible.

However, for certain analytes, closely matching SIC values between sample and standard is not critical. Some compounds, such as nitrate [1] exhibit stable measured isotope ratios across a wide range of concentrations. The same behavior is observed for acetate, where the isotope ratio remains unaffected by SIC intensity over most of the tested range [2]. In our measurements, although it was not always possible to perfectly match the SIC values between β-alanine in Murchison and the standard (Figure S1), the measured isotope ratios remained consistent with those obtained by conventional IRMS analyses (Figure 1). This suggests that for β-alanine, minor differences in SIC intensity did not compromise measurement accuracy under our analytical conditions. Nonetheless, the isotope linearity of amino acids has yet to be evaluated, and this should be addressed in future work, as achieving appropriate matching between extraterrestrial samples and reference standards can be challenging at very low analyte abundances.

**References**

[1] A. Hilkert *et al.*, “Exploring the Potential of Electrospray-Orbitrap for Stable Isotope Analysis Using Nitrate as a Model,” *Anal. Chem.*, vol. 93, no. 26, pp. 9139–9148, Jul. 2021, doi: 10.1021/acs.analchem.1c00944.

[2] E. P. Mueller, A. L. Sessions, P. E. Sauer, G. M. Weiss, and J. M. Eiler, “Simultaneous, High-Precision Measurements of δ^2^ H and δ^13^ C in Nanomole Quantities of Acetate Using Electrospray Ionization-Quadrupole-Orbitrap Mass Spectrometry,” *Anal. Chem.*, vol. 94, no. 2, pp. 1092–1100, Jan. 2022, doi: 10.1021/acs.analchem.1c04141.
